# Designing Virtual Natural Environments for Older Adults: Think-Aloud Study

**DOI:** 10.2196/40932

**Published:** 2023-04-07

**Authors:** Rikard Lundstedt, Johanna Persson, Carita Håkansson, Susanne Frennert, Mattias Wallergård

**Affiliations:** 1 Ergonomics & Aerosol Technology Department of Design Sciences Lund University Lund Sweden; 2 Division of Occupational and Environmental Medicine Lund University Lund Sweden

**Keywords:** virtual natural environments, user-centered design, qualitative method, real-time 3D graphics, older adults

## Abstract

**Background:**

Spending time in natural environments is beneficial for human health, but many older adults have limited or no access to natural environments. Virtual reality technology may be a means to facilitate nature experiences, and so, there is a need for knowledge on how to design virtual restorative natural environments for older adults.

**Objective:**

The aim of this study was to identify, implement, and test older adults’ preferences and ideas regarding virtual natural environments.

**Methods:**

A total of 14 older adults (mean age 75, SD 5.9 years) participated in an iterative process to design such an environment. We used think-aloud protocols and qualitative content analysis and established questionnaires that targeted usability, affective aspects, and side effects. These data guided the design decisions for incremental implementations of a prototype.

**Results:**

The participants’ preferences included trueness to reality in terms of rendition and behavior; traces of human activity and natural processes that trigger the imagination and provide believability; the ability to roam, explore, and interact with the environment; and a familiar, relatable environment that evokes memories. The iterative design process resulted in a prototype featuring many of the participants’ ideas and preferences, including a seated locomotion technique, animals, a boat ride, the discovery of a boat wreck, and apple picking. The questionnaire results indicated high perceived usability, interest, and enjoyment; low pressure and tension; moderate value and usefulness; and negligible side effects.

**Conclusions:**

We suggested 3 principles for virtual natural environments for older adults: realness, interactivity, and relatedness. Virtual natural environments should also provide a diversity of content and activities to accommodate the heterogeneity in older adults’ preferences. These results can contribute to a framework for designing virtual natural environments for older adults. However, these findings need to be tested and potentially revised in future studies.

## Introduction

### Background

Currently, there is much evidence that spending time in natural environments can be beneficial for cognitive function, mental health, and well-being [1-6]. Unfortunately, many older adults, who may be in particular need of such health benefits, have limited or no access to natural environments. For example, older adults living in residential care facilities may have a diminished ability to go outside because of limitations in functioning that are associated with old age. A few studies have suggested that replacements for real nature experiences such as indoor gardens can have positive effects [7,8]. A study found significant improvements in sleep, agitation, and cognition among 23 institutionalized patients with dementia who were allowed to cultivate and care for easy-to-grow edible plants indoors for 28 days [9]. Virtual reality (VR) has also been suggested as a way for older adults who cannot go outside to spend time in virtual natural environments (VNEs) [10,11].

A systematic review of indoor nature interventions (indoor gardens, plants, photographs, films, and 1 nonimmersive VR forest presented on a single large screen) for older adults in residential care settings found mixed results [12]. There was not sufficient evidence to recommend such interventions over other interventions or activities. In addition, interventions that involved photos, films, or nonimmersive VR were less effective than interactions with real forms of nature such as indoor gardening. However, immersive VR technology such as head-mounted displays (HMDs), where the viewer is completely surrounded by the virtual environment, has a higher potential to provide a sense of presence (ie, the perceptual illusion of *being there*) than nonimmersive media such as photos and single-screen displays [13-15]. Thus, although nonimmersive media would be unlikely to override the residents’ sense of indoor presence, a VNE experienced through immersive VR technology may provide residents with the perceptual illusion of being outdoors in nature.

A related study [16] compared different types of mediated nature experiences by depicting tropical coral reef scenes. The results showed that real-time 3D computer graphics via an HMD elicited a greater sense of presence, nature connectedness, and positive affect than filmed footage via a single screen. Another study comparing different delivery methods of psychotherapeutic interventions found reports of greater positive affect, satisfaction, and perceived credibility by participants exposed via an HMD than by those exposed via a single-screen display or using mental imagery [17]. Immersive VR has also been shown to induce physiological reactions in test participants similar to those induced by a corresponding real situation [18]. With this in mind, one can easily imagine the potential of VR to provide immersive experiences of simulated natural environments to care facility residents who have limited or no access to real natural environments. Recently, a few studies were carried out with older adults experiencing VNEs through immersive technology (HMDs). These studies reported some positive results, such as displayed enjoyment and relaxation [19] and positive responses, and that VNEs soothed and evoked memories [20] and improved positive affect and nature connectedness [21].

VNE studies often cite theories of restoration in natural environments [22], such as attention restoration theory [23], stress reduction theory [24], and the biophilia hypothesis [25]. Restoration theories can provide guidance for the design of restorative environments, for example, the 4 components by Kaplan [23]: *being away*, *extent*, *soft fascination,* and *compatibility*. Nukarinen et al [26] presented a framework connecting restoration theories and the measurement of health outcomes in VNE studies.

However, VR presents both limitations and possibilities that are different from those of a real natural environment. For example, VR cannot yet mimic the complexity, dynamic behavior, and immense detail of a real natural environment. On the other hand, it provides more or less complete control over the form and function of the virtual environment. Hence, a designer of VNEs is faced with choices that are not applicable in a real natural environment. Moreover, a user’s perception of an artifact is colored by the context in which it resides [27]. As VR provides a perceptual but not cognitive illusion [13]—that is, it feels real, but the user is aware that it is not—artifacts in VR are perceived differently from their corresponding objects in actual reality. As an example, a viewer may be impressed by how real a moss-covered rock looks in VR but may think nothing of a similar rock in the real world. Therefore, the knowledge of real restorative natural environments may not be applicable to virtual ones. To our knowledge, there are no frameworks, models, or guidelines for designing VNEs that are based on knowledge generated through a bottom-up approach in a VR context.

In general, older adults are seldom involved in participatory design processes; in particular, they are not involved in the development of new technologies such as VNEs [28]. As a result, older adults are rarely given a voice in the development of technological solutions aimed at them, which in turn may lead to the rejection or nonadoption of the technological solution in question [29].

### Research Questions

In this study, we aimed to identify, implement, and test preferences and ideas for VNEs by involving older adults in an iterative design process of a prototype. Thus, we explored the following research questions: (1) What preferences and ideas do older adults have for a VNE? and (2) How can we realize them?

We present our design process, the outcomes of 3 iterations, and some suggestions for what ought to be considered by designers when designing VNEs for older adults. We hope that our description of the explorative design process, along with interpretations and reflections on older adults’ preferences for VNEs, are a valuable contribution for designers and researchers alike and that our study may serve to indicate future directions in the development of VNEs for older adults.

## Methods

### Overview

**Figure 1:**
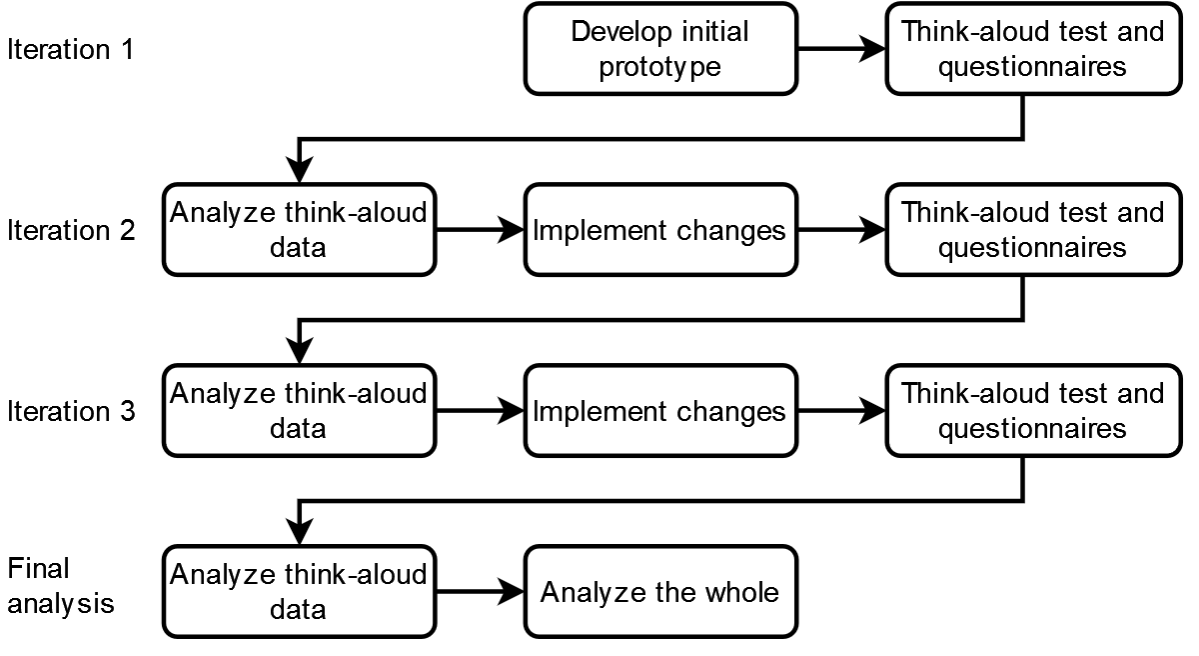
Overview of the iterative design process.

This study was conducted as a user-centered design process of a VNE to elicit the needs and preferences of users, challenge assumptions, and explore design ideas [30]. Users representing the target group were invited to iteratively test and provide feedback on the VNE prototype in a laboratory environment. Data were collected through a think-aloud protocol and questionnaires. Qualitative data were analyzed using an inductive qualitative content analysis method inspired by Graneheim and Lundman [31]. A total of 3 iterations were performed. The results of the data analyses of the previous iterations served to inform the design choices in the subsequent implementation phases. By iteratively developing the prototype and testing and analyzing the participants’ reactions (Figure 1), we were able to generate and test design concepts in small increments and, thus, build the VNE from the bottom up based on user input throughout the process. After completing the final iteration, we continued the analysis with a focus on the underlying threads of meaning running through all the data [31].

### Ethics Approval

Ethics approval for this study was obtained from the Regional Ethical Review Board, Lund, Sweden (2017/118).

### Test Setup

The tests were performed in a laboratory environment equipped with recording equipment for video and audio. The VNE prototype was developed and tested on a Windows PC with a GeForce GTX 1080 graphics card and an HTC Vive VR headset. We used the HTC Vive’s room-scale tracking capabilities and dedicated a *play area* of approximately 3 × 4 m within the laboratory.

Each test session comprised the following six steps (steps 1-3 were only included in the participants’ first sessions): (1) the participant was welcomed to the laboratory and given an explanation of the background of the study; (2) the participant was invited to undergo a short demonstration session to familiarize themselves with using the VR system; (3) the participant was asked to fill out a background questionnaire covering their previous experiences with VR and natural environments; (4) the participant underwent a concurrent think-aloud test session (described in the *Think-Aloud Protocol* section) while using the prototype (this session was video recorded); (5) the participant was asked to fill out questionnaires measuring usability, affective aspects, and side effects; and (6) the participant underwent a retrospective think-aloud session (described in the *Think-Aloud Protocol* section) while watching the recorded material from the concurrent think-aloud session (this session was also video recorded).

### Participants

A total of 14 participants (n=9, 64% women and n=5, 36% men) were recruited via retiree organizations in southern Sweden. Participants’ ages ranged from 69 to 90 years. The mean age was 75 (SD 5.9) years, and the median age was 73 years. The inclusion criteria were being aged >65 years, being able to speak and understand Swedish, having adequate eyesight to watch television, and being able to transport themselves to the laboratory. Exclusion criteria were any propensity for dizziness or motion sickness, dementia, reduced cognitive function, and problems with balance. The sampling was purposeful as the participants were intentionally selected to elucidate the VNE prototype from the perspective of an older person, and the sampling was convenient in the sense that the participants themselves chose to sign up for the study rather than being randomly selected [32].

In total, 57% (8/14) of the participants took part in the first iteration (Table 1). All of these participants (8/8, 100%) also took part in the second iteration along with 3 new individuals, resulting in 79% (11/14) of the total participants testing the second version of the VNE prototype. In the third iteration, 93% (13/14) of the total participants took part, whereof 23% (3/13) were new recruits and 23% (3/13) had participated in the second iteration.

**Table 1:** Overview of participants for each iteration in the design process.

Iteration	P1	P2	P3	P4	P5	P6	P7	P8	P9	P10	P11	P12	P13	P14
1	✓	✓	✓	✓	✓	✓	✓	✓						
2	✓	✓	✓	✓	✓	✓	✓	✓	✓	✓	✓			
3	✓	✓	✓	✓	✓	✓	✓		✓	✓	✓	✓	✓	✓

### Think-Aloud Protocol

**Figure 2:**
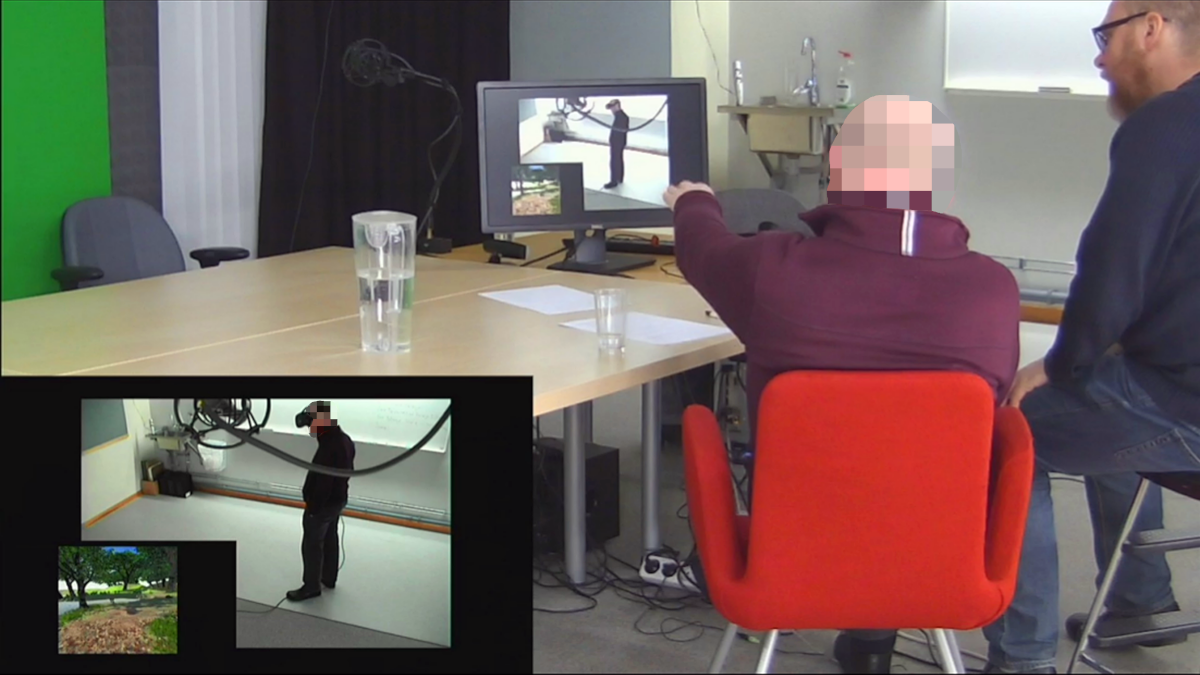
Image from the retrospective think-aloud session with one of the test participants.

During the test sessions, we used an adapted concurrent and retrospective think-aloud protocol [33]. Before each concurrent think-aloud session, the participant was asked to speak freely about their experience while using the VNE and share their ideas and suggestions for how they thought it should be changed or further developed. When necessary, the first author would use directive probing techniques [34] in an attempt to cover aspects of the VNE that the participant had not yet addressed, for example, by asking, “What is your perception of the water?” To allow the participant to go into greater depth in their reasoning, the first author would sometimes attempt nondirective probing techniques [34]. The retrospective think-aloud session was conducted in the same manner, only instead of using the VNE, the participant watched a video recording of the concurrent think-aloud session with the first author (Figure 2).

### Questionnaires

Although this was primarily a qualitative study, standardized questionnaires (Multimedia Appendix 1) were included in each iteration as a complement to gain an understanding of how our implementations affected the usability, affective aspects, and side effects of the prototype. Usability was measured using the *System Usability Scale* (SUS) [35], which is a widely used method for measuring the perceived usability of a system. It consists of 10 statements rated by the user on a 5-point Likert scale about a system’s characteristics, including complexity, ease of use, consistency, and learnability, for example, “I found the various functions in this system were well integrated.” Bangor et al [36] created the following adjective rating scale to interpret SUS scores: *Worst imaginable*, *Awful*, *Poor*, *OK*, *Good*, *Excellent*, and *Best imaginable*. Affective aspects were measured using the *Intrinsic Motivation Inventory* (IMI) [37], which is a multidimensional measure that can be used for assessing a person’s experience of an activity. The IMI can be customized to only include the dimensions relevant to a particular use. We chose to include the following dimensions: interest and enjoyment, value and usefulness, and felt pressure and tension. We chose to exclude the following dimensions: perceived competence, effort, and perceived choice. Side effects were measured using the *Virtual Reality Symptom Questionnaire* [38]. The questionnaire consists of 13 questions measuring physical symptoms that may be experienced when using a VR system, such as headache or nausea, rated by the user on a 7-point scale. In their first test sessions, the participants were also asked to fill out a background questionnaire regarding their past and present experiences of natural environments, their previous level of experience with VR, and their state of well-being at the moment.

### Analysis

After each iteration, the notes from the think-aloud sessions were analyzed by one of the authors (RL) using the recordings as a reference when necessary. Each idea, preference, opinion, suggestion, or other thought expressed by the participants was coded using an inductive content analysis method inspired by Graneheim and Lundman [31]. By searching for patterns within the codes, categories emerged that guided the decision-making when implementing new functions, content, and other changes in the VNE prototype.

In the implementation stages of each iteration, authors RL and MW discussed the categories (hereinafter referred to as *preferences*) to reach an agreement on how they should be interpreted from a design perspective, that is, how the preferences could be realized as changes to the prototype. RL then implemented the agreed-upon changes. As the participants reacted to the new features in the subsequent tests, the preferences that had been realized within the prototype were to some extent validated by the participants themselves. They would sometimes build on and develop them through more specific requests, reflections, suggestions, or ideas. Thus, there was a progression and deepening of ideas over the course of the iterations. We attempted to reflect this in this paper by presenting the preferences in the order in which they were realized.

After completing the final iteration, we continued the analysis assuming a bird’s-eye view of the data. By reflecting on the patterns and interrelationships of the preferences, we arrived at a set of principles for VNEs for older adults. All the authors reached a consensus on the principles by means of repeated discussion.

### The Initial Prototype

**Figure 3:**
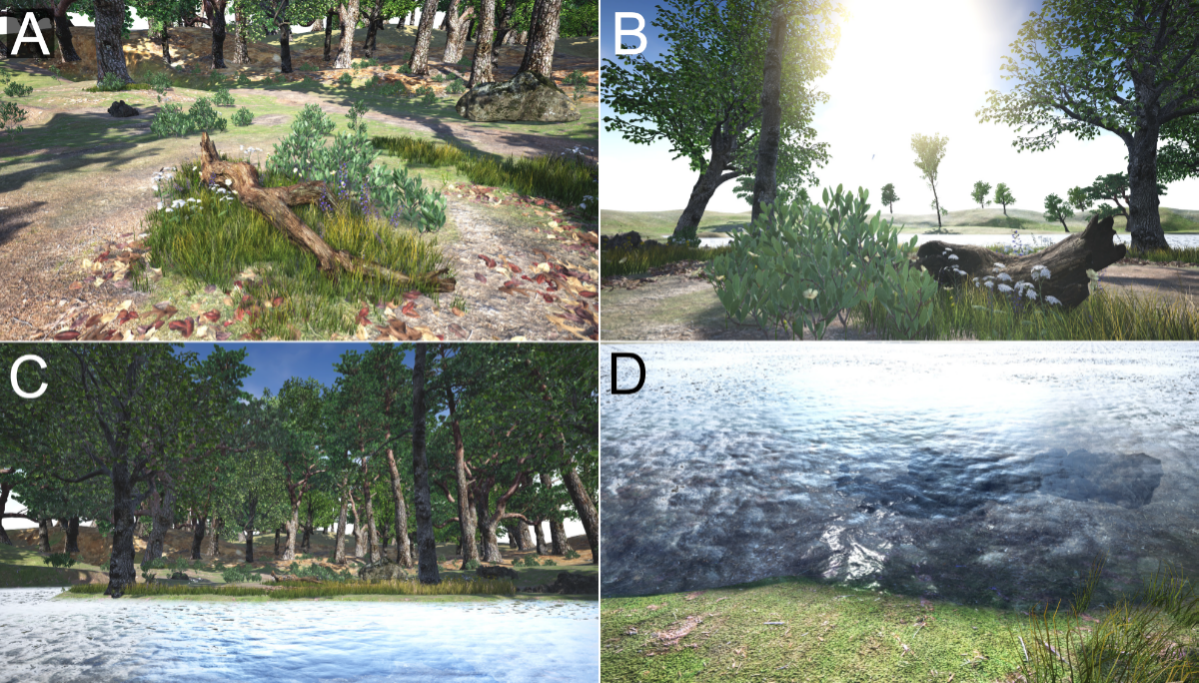
Screenshots from the initial prototype: (A) detail of the ground, (B) view facing the lake, (C) view of the play area from the lake, and (D) detail of the water.

In the construction of the VNE prototype, we used computer-generated real-time 3D graphics via the *Unreal Engine* (version 4; Epic Games, Inc) game engine. Thus, it generated visuals, audio, and other potential sensory outputs in the moment based on user input and programmed behavior. The virtual environment was a compound of authored and sampled (from the real world) materials such as 3D models, images, and sound files. To leave room for the participants’ ideas, we designed the initial prototype to be a rather bare and simple starting point. As the subject of this study was the limited access to natural environments in one’s own region, the features included in the scene were of the type that one might expect to see in the geographical region in which the study was set. After putting on the HMD, the participants would find themselves next to a lake near a forest (Figure 3) in a scene comprising various 3D models of grass, moss, water, rocks, sand, dirt, trees, leaves, flowers, and natural debris such as old twigs and logs. Other than the water and grass, which were animated to simulate movement caused by the wind, all objects were static. The environment was lit like a sunny summer day, and the sounds of small birds could be heard. The participants entered the simulation standing up and were able to move around freely over the play area using their own bodies as they would in the real world. This freedom to move allowed the participants to turn around and obtain a complete 360° view of the scene, inspect details such as flowers on the ground by moving closer to them, and look behind objects such as rocks and trees.

## Results

### Overview

The average concurrent and retrospective think-aloud sessions lasted 29 and 23 minutes, respectively. Analyses of the sessions yielded several categories reflecting various topics and containing both the participants’ opinions of the current iteration of the VNE along with ideas, preferences, and suggestions for future versions. These were not necessarily exclusive to a particular iteration; however, we present them in an order that shows the progression of the design process. Hence, for each iteration, we present the ideas, preferences, and suggestions that inspired the implementations made in that particular iteration or that are linked to it in other ways. By doing so, we aimed to show how participation guided the design rather than being tokenistic.

### Participants

The participants generally had very little experience with VR but much access to and experience of spending time in natural environments (Table 2). All except 1 of the 14 participants (13/14, 93%) answered “yes” to the question “Do you have access to a garden in connection with your home?” Generally, the participants reported high perceived well-being at the moment. Some background questions were left out of this table.

**Table 2:** Questions and scores from the participant background questionnaires.

Question	Mean score (SD)	Median score
How much experience do you have with using VR^a^? (1=none; 5=very much)	1.1 (0.4)	1
How much experience do you have of natural environments? (1=none; 5=very much)	3.9 (0.9)	4
How great are your possibilities to spend time in natural environments? (1=none; 5=very great)	3.9 (1.1)	4
How often do you spend time in natural environments? (1=never; 5=very often)	3.2 (0.8)	3
How do you feel? (1=very bad; 5=perfectly good)	4.3 (0.8)	4.5

^a^VR: virtual reality.

### The First Iteration

**Figure 4:**
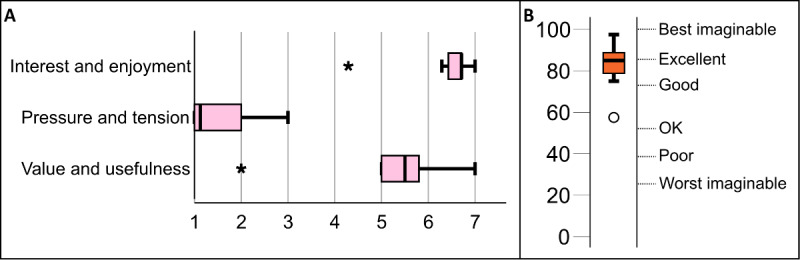
(A) Intrinsic Motivation Inventory and (B) System Usability Scale results for the initial prototype in the first iteration.

During the first iteration, the participants tested the initial prototype, spoke their preferences and ideas during the think-aloud sessions, and filled out the questionnaires. Figure 4 shows the results of the IMI (Figure 4A) and SUS (Figure 4B) questionnaires. The IMI scores indicated that most participants (7/8, 88%) found the experience to be highly interesting and enjoyable and somewhat useful and valuable to them and did not feel much pressure or tension. The median SUS score was 85, which is just below *excellent* on the adjective scale by Bangor et al [36]. An analysis of the think-aloud data is presented in the following section.

### The Second Iteration

To begin the second iteration, we analyzed the think-aloud data from the first iteration. Table 3 presents the participants’ preferences along with suggestions and illustrative quotes from the participants. As can be seen, they preferred more movement, life, and change and the ability to roam and explore the environment but also sit and relax, and they emphasized the importance of authenticity and realism.

**Table 3:** Participants’ preferences and suggestions from the think-aloud session in the first iteration.

Preferences	Description	Participants’ suggestions	Illustrative quotes
Movement, life, and change	The environment was perceived as static and sterile and lacked life, change, and movement.	Add animals, changes in the light from cloud movements, and movement in the trees from the wind.	“The nature looks like a still [photograph].” [P4] “In [real] nature, there is some movement in some way all the time...you see birds, but you don’t see any birds here.” [P5] “I think it would be positive with something that showed up. There could come a hedgehog, and butterflies, and a bird that flew.” [P3]
Roaming and exploring	Participants wanted to move beyond the play area and roam and explore the environment.	—^a^	“You feel a little caged in, I must say.” [P7] “I would have liked to go a little further into the forest, but I couldn’t.” [P8] “You want to check out what is behind the hill there.” [P2]
Sitting, passive enjoyment, and relaxation	Participants wanted to sit and relax in the environment, enjoying the view.	Add the possibility to sit on a recliner, bench, blanket on the ground, or in a boat on the water and have a picnic.	“I would like to be able to sit down and just sit and look and listen to birds singing, not do anything other than just relax.” [P5] “I would like to have something to sit on with a view of the water and the flowers.” [P7]
Authenticity and realism	In various ways, participants emphasized the importance of authenticity and realism in the VNE^b^.	There should be movement in the trees from the wind just as there is in the grass; otherwise, it is inconsistent.	“The trees look a little like trees on a model railway.” [P5] “I think you need a rather high degree of realism.” [P7] “It should be flowers that exist in reality.” [P1] “I would have had difficulties [relaxing in an inconsistent environment]...because then I focus on that [the inconsistencies].” [P6]

^a^Not available.

^b^VNE: virtual natural environment.

To continue with the next step in the second iteration, we proceeded to interpret the participants’ preferences and suggestions in terms of how we could realize them as changes to the prototype. Table 4 presents the implementations along with the preferences they addressed and the reactions of the participants. We added various animals, movement of the trees, the possibility to roam and explore the environment, and a jetty on the lake. As can be seen, we implemented 2 different ways to enable the participants to roam and explore the environment. Although teleportation is commonplace in many VR applications, the experience of teleporting is fundamentally different from walking as one does not perceive any gradual movement, and participants had specifically expressed a wish to take walks. Therefore, we realized the need for a technique closer to walking. An obvious solution was to simply implement forward propulsion in the direction of the forward vector of either the HMD or the handheld controller at the push of a button on the controller. However, many older people are wheelchair users or experience reduced postural stability and may lose their balance and fall because of vection. We speculated that there would be very few residents at care facilities who could manage immersive VR while standing up. We also considered that a seated locomotion technique might accommodate to some degree the participants’ preference for sitting, passive enjoyment, and relaxation.

**Figure 5:**
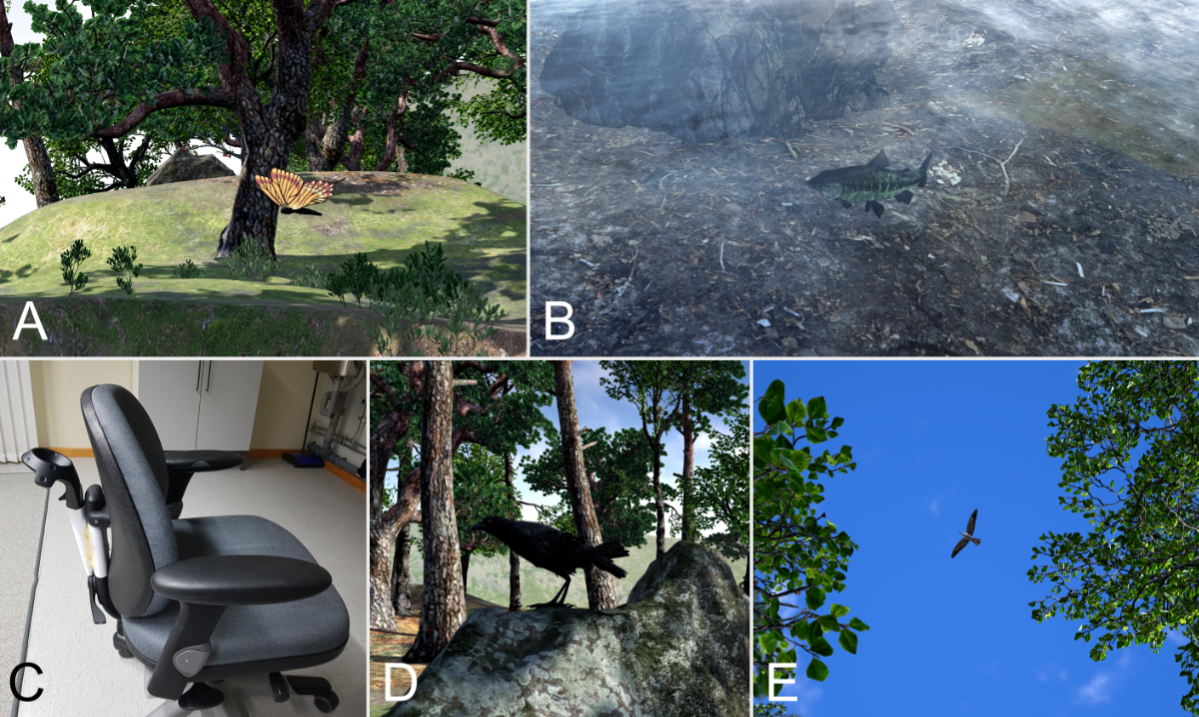
Implementations of the second iteration: (A) screenshot of a butterfly, (B) screenshot of a fish, (C) photo of the physical swivel chair, and (D) and (E) screenshots of birds.

In light of this reasoning, we implemented a system in which a user could sit down while driving around in the virtual world. This was accomplished by fastening one of the handheld controllers to the back of a swivel chair, allowing the chair’s position and orientation to be tracked by the VR application (Figure 5). In the virtual environment, the chair was represented by a simple 3D model. The virtual chair’s position and orientation were updated in real time to correspond to those of the real chair. To control the throttle, the user could press a button on the other handheld controller while sitting in the swivel chair, which would result in them experiencing forward propulsion in the direction of the chair in the virtual environment. To steer, the user would simply turn the chair in the direction in which they wanted to go using their feet. Thus, the user would not experience circular vection while steering, something that is associated with motion sickness [39].

**Figure 6:**
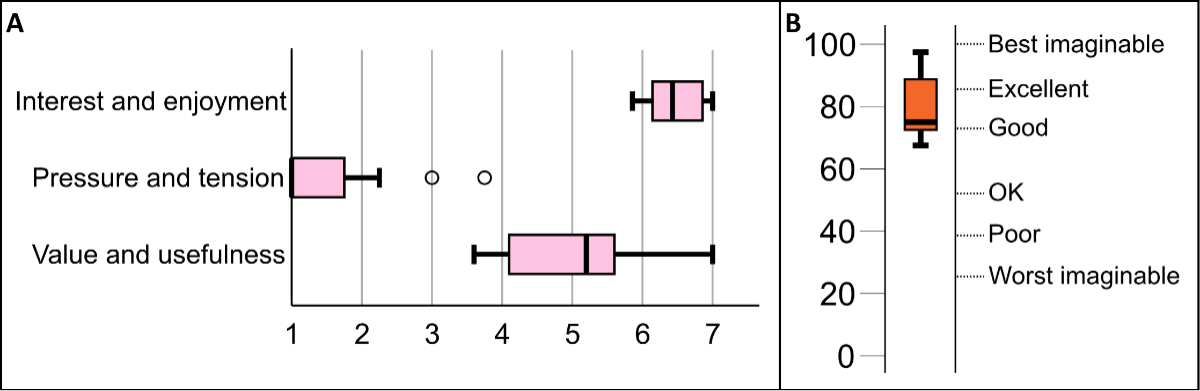
(A) Intrinsic Motivation Inventory and (B) System Usability Scale results for the second iteration.

At the end of the second iteration, the participants tested the new prototype while thinking aloud, reacting to the new implementations and providing new preferences and suggestions. As before, they filled out the questionnaires. Table 4 presents some quotes that illustrate the participants’ reactions. Figure 6 shows the IMI (Figure 6A) and SUS (Figure 6B) scores. The IMI scores indicated that the participants found the experience of the new VNE to be highly enjoyable and interesting, that they did not feel too pressured or tense, and that the value and usefulness of the experience to them was neutral to high. The median SUS score was just above *good* on the adjective scale by Bangor et al [36]. This indicated that, although the complexity of use increased with the implementation of teleportation and the swivel-chair vehicle, usability remained satisfactory.

**Table 4:** Realization of participants’ preferences and their reactions.

Implementations	Addressed preferences	Description	Reactions of the participants
Animals	Movement, life, and change; authenticity and realism	Birds, fish, and a butterfly exhibiting natural-like behavior controlled by AI^a^ scripts	“It’s considerably more natural, especially with the butterflies and birds.” [P6] “It feels more alive, it doesn’t feel as artificial.” [P5] “I thought it was positive with the butterfly and the birds, and the fish. It became more alive.” [P1] “It must be a different country because such fish we don’t have here.” [P9]
Movement of the tree branches from the wind	Movement, life, and change; authenticity and realism	Animation of the tree branches to resemble movement from the wind	“It’s good that it moves a little; it feels considerably more natural.” [P5] “The branches move a little. They didn’t do that last time. That’s nice. So that it’s something more that happens.” [P3] “This branch over here moves but the trees over there do not...when you notice that, you feel that something is not right.” [P4]
Teleportation	Roaming and exploring	Ability to instantly teleport oneself by aiming the handheld controller to an arbitrary point in the environment and pressing a button	“This was a boost, absolutely. You become more active; you don’t simply stand and look around.” [P4] “It feels more free.” [P8] “It becomes considerably nicer than to be stuck in one place. You get more experiences.” [P5] “It feels very artificial, that way to move. You are somewhat in a computer game context.” [P11]
Swivel-chair vehicle	Roaming and exploring; sitting, passive enjoyment, and relaxation	Ability to drive in the environment while sitting in a swivel chair, press a controller button to instigate propulsion, and steer by turning oneself in the desired direction	“This is an amazing feeling. It feels like the chair is moving. This was cool.” [P4] “Here I could move where I wanted, and see that I moved.” [P8] “It is no problem to handle this.” [P7] “I think it’s very awesome, but it is not natural for me to move like this [because I’m not a wheelchair user].” [P5] “It doesn’t go very fast. Can you increase the speed a little?” [P11]
Jetty	Roaming and exploring; movement, life, and change	A jetty to accommodate exploring the lake and observing the fish	“It was very interesting to go out on the jetty and look down into the water.” [P9] “It’s fun to walk out on the jetty; you see the fish better from the jetty.” [P7] “I think this is rather fascinating, to stand and look down.” [P11]

^a^AI: artificial intelligence.

### The Third Iteration

Table 5 presents the participants’ preferences and suggestions that were expressed during the think-aloud sessions of the previous iteration (second iteration). Preferences that emerged included bodies of water; the ability to look at, study, or inspect things; a rich diversity of activities; and human presence or activity.

**Figure 7:**
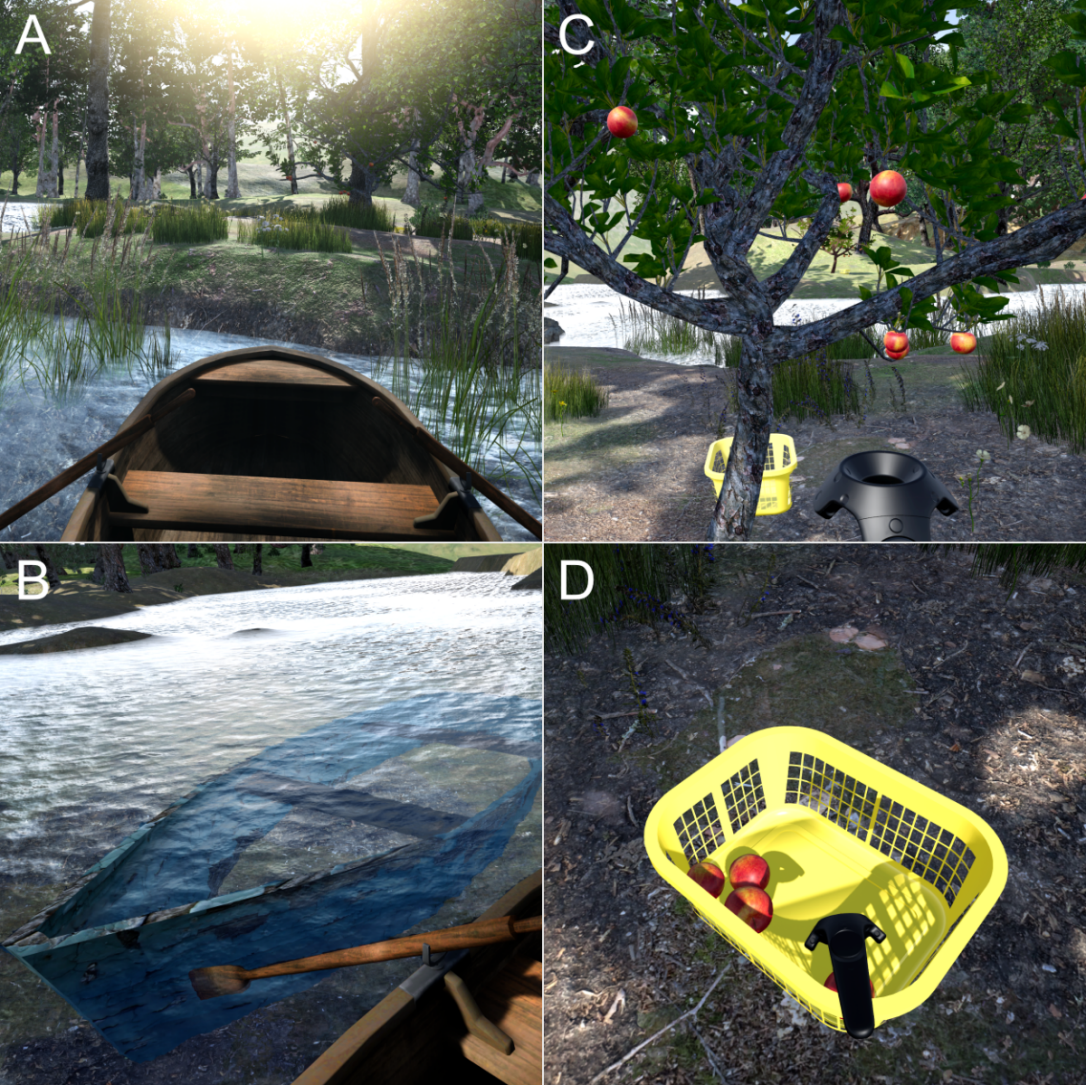
Implementations of the third iteration from the point of view of the participants while in the virtual natural environment: (A) driving the rowboat, (B) discovering the sunken boat, and (C) and (D) picking apples from the apple tree and placing them in the basket.

We proceeded to analyze how the participants’ new preferences could be realized and made additions and changes to the prototype accordingly. Table 6 presents these implementations, the preferences they were meant to address, and some of the participants’ reactions that were gathered during the subsequent think-aloud sessions that concluded the third iteration. As shown in Figure 7, the implementations consisted of a small rowboat and a peninsula (Figure 7A), a sunken boat (Figure 7B), and an apple tree (Figures 7C and Figure 7D).

**Figure 8:**
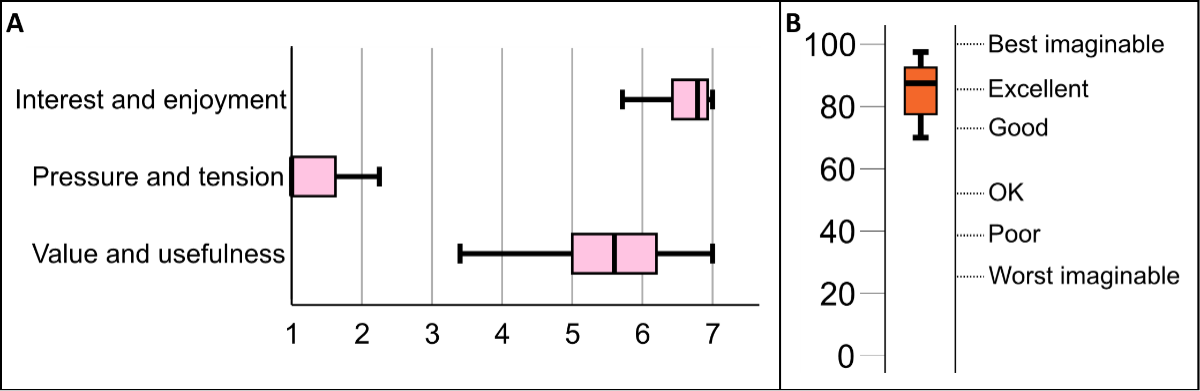
(A) Intrinsic Motivation Inventory and (B) System Usability Scale results for the third iteration.

Figure 8 shows the IMI (Figure 8A) and SUS (Figure 8B) scores for the third iteration. The IMI scores indicated that the participants found the experience highly interesting and enjoyable and did not feel much pressure or tension and that their view of its value and usefulness to them was neutral to high. The median SUS score was just above *excellent* on the adjective scale by Bangor et al [36], which again indicated that, despite the increased complexity of use that was introduced with the rowboat and the apple tree, the participants viewed the prototype as highly usable.

**Table 5:** Participants’ preferences and suggestions from the think-aloud session in the second iteration.

Preferences	Description	Participants’ suggestions	Illustrative quotes
Water	Participants expressed a preference for bodies of water and for being in or on the water.	Add the possibility to ride in or drive a boat such as a rowboat or canoe.	“I was actually delighted by the lake, especially with the translucency [so that] you could see the fish, and that you could drive out in it.” [P11] “I think you could preferably have a boat to step into...and then you could glide out on the lake.” [P1]
Interactivity	Participants had many diverse preferences for activities in the VNE^a^.	Add the ability to pick things such as flowers, mushrooms, apples, or shells; go fishing, canoeing, and mountain climbing; and read a book, grill, swim, fly a kite, and play with their grandchildren.	“You can imagine an apple tree, you pick apples.” [P4] “One should be able to pick some flowers.” [P3] “If you go out on the jetty, you could fish.” [P9]
Look at, study, or inspect things	Participants wanted to look at, study, or inspect interesting things in the environment.	Provide the possibility to look down into the water and see things such as clams, crabs, aquatic plants, or just the bottom; inspect birds closely; and look through binoculars.	“[Y]ou stop if there is something that you find interesting...and you think ‘exciting,’ and I want to see what that looks like...you want to inspect it closer.” [P10] “That there was something on the bottom, to look [at] and contemplate, fish, and it can be whatever, rocks, clams.” [P8]
Human presence or activity	Participants thought that one should be able to see or hear humans, human activity, or traces thereof.	People who are visibly present in the distance, walking by, swimming, or working in a garden; occasional sounds from agriculture, forestry, or a car in the distance; an airplane in the sky; and items or structures that reveal human presence, such as a bench, a fireplace, an old bicycle, or an old boat	“There are no people around. It is very empty of people.” [P1] “[A] stone bench over by the beach somewhere, that you can imagine where people have sat and enjoyed themselves.” [P7] “You could see some pollution, an old bicycle. Something you don’t expect to see.” [P6]

^a^VNE: virtual natural environment.

**Table 6:** Realization of participants’ preferences and their reactions.

Implementations	Addressed preferences	Description	Reactions of the participants
Small rowboat	Water; look at, study, or inspect things; interactivity; human presence or activity	A small rowboat that a user could enter and drive on the water	“It was very nice, cozy in some way, and as if I had rowed out myself. It feels natural.” [P13] “It’s rather fascinating to look down into the water. It looks rather true to nature. There comes a little fish. It moves like a fish should.” [P4] “It’s a little weird that it moves without you rowing. It feels like you had had a small outboard motor.” [P11] “The reeds should move out of the way [when driving the boat over them].” [P6]
Sunken boat	Look at, study, or inspect things; human presence or activity	A sunken boat that could be discovered by driving past in the rowboat	“It’s exciting because you didn’t see that it was a boat until you got closer.” [P9] “It’s good that things happen; that there are things...to discover...I think [it] makes it a little more interesting.” [P10] “You see that, gee, here is something. I must inspect it further.” [P5] “It also provides the feeling that there are people.” [P11]
Peninsula	Realism and authenticity; roaming and exploring	Reshaping of the landscape to form a peninsula in the lake; adding more trees and flowers with the intention of making the environment look more authentic and natural and more interesting to explore	“[I]t looks much more natural, trees and such. It’s not as artificial as the first time.” [P5] “It became a more intimate landscape. It was like a desert before.” [P2] “I think it [the lake] is more natural now. It looked small and landscaped in the beginning.” [P1] “It is considerably more alive, more to discover.” [P4] “I don’t experience the ground as natural.” [P7]
Apple tree	Interactivity; human presence or activity	An apple tree from which users could pick apples and place them in a basket	“This is a lot of fun.” [P12] “I think it’s good that you can do something; that you can drive in the boat, pick apples, walk around a little; that it doesn’t become just a passive experience.” [P5] “You feel involved, active; that you can do something yourself.” [P1] “I affect something [in the environment]. It enhances the experience.” [P4] “You can imagine that there was a farm here before and that an apple tree remains.” [P2] “It becomes unrealistic because if I pick berries, I want to be able to eat them.” [P10]

### Final Analysis

Upon completion of the third iteration, we proceeded to analyze the latest round of think-aloud sessions. Table 7 presents both preferences that relate to the latest implementations and those that surfaced throughout the study that did not pertain to any particular iteration or implementation. Participants reflected that discovering traces of human activity triggered their imagination. Generally, participants preferred the environment to be familiar and relatable. Other ideas that surfaced recurringly throughout the study were the ability to eat or drink, that the sound should be more realistic and varied, and being able to touch and smell the environment and feel the wind.

**Table 7:** Participants’ preferences and suggestions in the think-aloud session in the third iteration.

Preferences	Description	Participants’ suggestions	Illustrative quotes
Discovering traces of human activity triggers the imagination.	Participants reflected that traces of human presence or activity made the environment more interesting and set their imaginations in motion. They wondered what events had taken place, which people had been there before, and what had happened to them.	Add more traces to discover a cairn, an old pot, ruins of an old house, traces of a garden, an old well, a root cellar, or an item that someone had lost or dropped. Make it so things change in between sessions—someone picked all the apples or hauled away the shipwreck.	“You imagine what has happened to the people who lived here.” [P9] “It sets the imagination in motion...You begin to wonder. It’s positive compared to nature that is completely free from human traces.” [P3] “The little shipwreck can be gone the next time. Then you discover that someone has taken care of it, because it’s not so good that it lies there and rots.” [P1]
Familiarity and reminiscence	Participants preferred the environment to be familiar and to be able to identify with it and relate to memories, such as from their childhood.	Make it possible to carry out activities one did as a child.	“Something that you recognize; an environment like you grew up in.” [P8] “An older person wants to recognize themselves [in the environment].” [P7] “It’s quite nice if you can identify [species] so you can say, ‘Wow, it’s a great tit, or a blue tit.’” [P3]
Eating or drinking and socializing	Participants expressed a desire to eat or drink something, potentially in the form of a social situation such as having coffee together with others present in the VNE^a^ at the same time.	The possibility to have a picnic on a blanket on the ground, a wooden bench table, or a cafeteria	“You could have coffee out here...Suppose that there...is one more [person] who in some way interacts...perhaps sets the table, pours coffee, says something perhaps, ‘Come now it’s time for coffee.’” [P11] “I can imagine sitting on one of these green slopes and having coffee or a little picnic.” [P14]
Sound	Participants thought that sounds that realistically should be there were missing and that there should be more variation.	Sounds from the wind, rustling when walking over fallen leaves, and splashing of water when driving the boat; sounds that bring variation: a cuckoo or pigeon, a car in the distance, or people walking	“I would have liked to hear splashing [while driving the boat]. It would have strengthened the illusion.” [P5] “You could hear a sound in the background; a tractor, or a boat on the lake, some momentary sounds.” [P2]
Other modalities	Participants wanted to experience the environment through additional senses.	To feel the breeze and the warmth of the sun; touching things, trees, rocks, or apples; smelling things, flowers, or coffee; having feet lowered into a bucket of water while being in the virtual water	“You would like to touch them [the trees] even though you know that there is nothing. If I get really close to it, like this, you want to touch it. But then there is nothing.” [P10] “Wind is probably the most important that you can feel...so that you feel like you are outside.” [P1] “If I’m out in nature there are often smells. That I don’t sense here.” [P11]

^a^VNE: virtual natural environment.

### Observations of Usability

Although the SUS scores indicated high usability throughout the study, in our observations of the participants exploring the environment, we noticed that some had difficulties finding the correct buttons on the controller for teleporting and driving. Sometimes, they teleported by mistake by accidentally pushing the teleport button. The task of approaching the apple tree and ending up in a location convenient for picking apples seemed difficult at times. This was because the participants often came too close to the tree or even moved inside it. The participants often flinched when they came too close to the branches.

### VR Symptoms

The Virtual Reality Symptom Questionnaire revealed very few symptoms, with means of <1 on a scale from 0 to 6 on every symptom measured in all 3 iterations. However, there were isolated medium-high scores reported: a score of 3 for dizziness in iteration 2 and 2 scores of 4 for blurred vision in iterations 1 and 2.

### Principles of a Meaningful VNE

#### Overview

As a final step in the process, we took a bird’s-eye view of all the participants’ preferences to reveal recurring threads of meaning and condense the findings into applicable principles. By reflecting on the patterns and interrelationships of the preferences, 3 main principles emerged—realness, interactivity, and relatedness. These could be considered by designers of future VNEs. However, they need to be tested and potentially revised in future studies.

#### Realness

Realness refers to how complete the experience is in terms of presence, realism, and believability. A VNE should provide a sense of presence (ie, a sense of being in place). This can be accomplished by providing (approximate) real-world responses to actions [40]. Sideways head movements, for example, should allow the user to look behind objects, and touching objects should optimally provide haptic feedback:

It would be very interesting to sense that feeling [touch the rock]...For me, it would be very positive. Because then I am absolutely in nature.

P6

A VNE should provide believability. By this we mean a sense of a complete world that does not end behind a backdrop—a world in which there are interesting things to discover, such as animal and plant life, traces of human activity, details that reveal natural processes, and distant sounds. We propose that these characteristics can contribute to a feeling of a complex living world that continues beyond the reach of one’s eye and stretches back into a historical past:

It’s a very static environment. I must say that I don’t feel like I’m out in nature for real, more like I’m standing on a stage with backdrops.

P11

It’s more alive, just that little butterfly alone contributes a lot, I must say.

P1

[H]ere has been a house, and there you can see an old apple tree...it triggers the imagination, that someone has lived here and how were they doing? How were they able to live here in the middle of nowhere? Such things can also contribute a little.

P5

It provides realism to it all. I mean, it’s certainly not unusual that there lies an old miserable-looking boat by the shoreline.

P14

The trees look too nice...there are none [branches] that has been broken off by the wind and such.

P5

A VNE should provide a sense of trueness to reality, such as accuracy in rendition and the combination and behavior of natural and human-made elements:

It should be flowers that exist in reality.

P1

I don’t expect to see an apple tree in a pine forest.

P11

You stop enjoying it, lose interest in it...when you go into the details and it is incorrect. Then it’s maybe a little lesser of an experience.

P9

#### Interactivity

In addition to basic VR sensory-motor contingencies—including moving one’s body, head, and eyes to change gaze direction and looking behind or under things—a VNE should preferably provide the possibility to engage in activities (eg, exploring the environment and picking mushrooms). Activities should be congruent with the users’ capabilities, interests, and identities. Care should be taken to also accommodate for passive activities (eg, to just sit and relax looking at things). As previously stated, user actions should elicit responses that are as close to reality as possible:

To be able to do different things, walk around, look down into the water properly, drive the boat, pick apples. They are positive elements in it compared to the first time, [which was] just a quiet and rather flat environment. Suddenly a lot of things happen, you can do a lot of things.

P5

It was a lot of fun to pick the apples and put them in the basket. It was an extra-experience...because you do something, and you see that it works. But, if you drop an apple, normally, “thud” it says, but these were very soft.

P13

#### Relatedness

A VNE should enable a user to identify with it and relate to memories (eg, the environment should be familiar, and the vegetation and animals should be of species that one can recognize):

[It should be an environment] that you recognize yourself in, that you can relate to.

P3

Where I used to walk when I was a child, for example, I think would be a very nice experience...Because then you get it related directly to yourself.

P13

I have some good friends who have a family farm by a small lake...I can imagine that if they saw this, it would evoke memories in them in their old age; but to evoke such memories in me, it should be by the sea.

P10

To me, when it comes to nature...you relate to the memories that you have from nature, and if it matches better, it feels like it’s more natural.

P8

## Discussion

### Principal Findings

This study was conducted to investigate older adults’ preferences and ideas regarding VNEs and how these can be realized. To our knowledge, this is the only study in which such preferences for VNEs were collected through an iterative participatory design process using an inductive approach. Our results suggest the preferences outlined in Textbox 1.

It should be noted that preferences for activities were very diverse among our participants. Overall, we propose that one should pay attention to multiple choices of activities and variations of features. Ideally, a VNE should be tailored to the individual.

The participants’ responses to the questionnaires and reactions to our implementations suggest feasible ways to realize some of the preferences for VNEs that emerged during this study (Textbox 2).

**Participant preferences.**

**Preferences for realness**

- Trueness to reality in the rendition and behavior of natural and human elements (eg, accuracy in prevalence and combination of species)
- Real-world responses to actions, such as accurate audial and visual feedback (eg, displacement of objects and sound of impact)
- Extended range of sensory modalities such as touch, smell, and temperature

**Preferences for interactivity**

- The possibility to roam and explore the environment and inspect interesting things such as natural and human-made elements
- The possibility to engage in activities such as picking mushrooms, taking a boat ride, or having a picnic
- The possibility to sit and just relax observing the environment

**Preferences for relatedness**

- A familiar, relatable environment in which one can recognize oneself
- Being able to recognize and identify species and natural features
- Being able to relate to memories

**Feasible ways to realize participant preferences.**

**Realizations of realness**

- The occurrence of animals (eg, birds, fish, and insects) and the movement of vegetation and other natural features (eg, from wind) make the environment appear more alive and real.
- Traces of human activity trigger the imagination, allowing users to picture people and past events in the environment. This contributes to their perception of the environment as a real place.

**Realizations of interactivity**

- The possibility to roam and explore the environment enhances the experience of a virtual natural environment. It can activate and provide a sense of increased freedom to users.
- Teleportation and forward propulsion with seated swivel chair–directed steering are feasible methods for roaming and exploring the environment. However, they can be perceived as unnatural in their own ways.
- Activities that involve some manipulation of the environment, such as picking apples, can enhance the experience, making users feel involved and active. However, unrealistic mechanics of activities may be unfavorable for the experience.

Some of our results showed that participants wanted to be active in the VNE. They wanted to explore, inspect, and interact with the environment (eg, pick the flowers, touch the rocks, and feel the water). At the same time, they wanted their actions to be true to reality. This is not so surprising as perception requires action [41]. For example, shifting one’s head sideways enables one to see behind things. According to Slater et al [40], VR works by providing real-world responses to actions. That is how one acquires a sense of presence, of being in place in the virtual environment. If one performs an action and there is no response or the response is too far from reality, one will momentarily break presence. Thus, consistent with our findings, we propose that the possibility of interaction (that approximates real-world interaction) is important in VNEs. Although this proposal may seem obvious, many studies of VNEs use technologies that feature minimal or no interaction, such as 360° video [26], which does not support sideways head movements. However, 360° video has other advantages, such as the ability to capture existing environments, which can make the participants’ preference for relatedness (ie, familiar environments and the possibility to relate to memories) more economical and feasible to realize. Orr et al [20] used 360° video to provide VR experiences of local beaches to older adults with mild to moderate cognitive or memory impairment. They found that familiarity with the environments brought the participants enjoyment in identifying places and relating to memories. They also found indications that the VR provided was “sufficiently credible” and that participants were able to acquire a sense of presence. However, the presence of other (filmed) people in the 360° videos provoked in the participants a desire to interact, which, naturally, was impossible. A recent study of VNE use by patients with breast cancer advised that future studies should “focus on activities that encourage connection with nature (rather than simply exposure to nature)” [42].

The biophilia hypothesis argues that humans have a biologically based need for a sense of belonging to the natural world. This connectedness with nature is instrumental for human well-being according to the hypothesis [25]. One can speculate that our participants’ preference for being able to identify with and relate to the environment is partly an expression of nature connectedness, and interaction with the environment has the potential to promote connectedness and relatedness. Other studies have measured nature connectedness in VNEs. One study [16] found higher nature connectedness in participants using an immersive VNE than in those watching a corresponding natural environment on a television screen. Another study [21] suggested that nature connectedness mediated positive affect in older adults using an immersive VNE.

On the basis of the participants’ preferences and reactions to our implementations, we propose that a VNE for older adults should be true to reality in rendition and behavior and that traces of human activity and natural processes can promote the perception of it as a real place. This is very reminiscent of what Slater et al [40] present as a contributing factor to *plausibility* (Psi), which is the illusion that the events happening in the virtual environment are actually taking place. In addition to trueness to reality in appearance and behavior, Psi relies on the virtual environment in some way acknowledging the user (eg, that a virtual human character plausibly responds to an action by the user). Although this study did not involve social interaction with virtual human characters, we found that our proposal is congruent with the presentation of Psi by Slater et al [40].

### Limitations and Future Research

The focus of this study leaned heavily toward gathering the participants’ ideas, preferences, and suggestions and revealing unforeseen problems and considerations in the process of designing a VNE. This focus was a determining factor in the choice to include the think-aloud protocols in the study design. There is a risk that the extensive recurring think-aloud sessions generated bias within the participants, which could have affected the scoring of the questionnaires. It is not difficult to imagine that the participants may have adopted a positive stance as they had someone who was listening carefully to them and was genuinely interested in their opinions, as well as experiencing firsthand that their opinions had an impact on the design of an important innovation. As the participants generally had very little experience with VR (Table 2), its novelty could have contributed to the high IMI scores. The findings of this study need to be tested and potentially revised in future studies.

As is evident from the inclusion and exclusion criteria and the results of the background questionnaires, the participants in this study were relatively healthy and mobile and had access to natural environments. Thus, they differed in many ways from the proposed group that this study is aimed at, namely, residents of residential care facilities who have limited or no access to natural environments because of ill health. The lower scores the participants gave on the value and usefulness dimension of the IMI questionnaire compared with the other dimensions may be a testament to this fact—they simply may not have found the VNE very useful as they were able to visit real natural environments. This study was also carried out in a controlled laboratory environment and so did not consider the complexity of residential care facilities and the network of different stakeholders involved. Consequently, the next step would be to continue these research efforts with residents and staff at care facilities in the real world.

The HTC Vive has a setting to adjust the distance between the lenses to match a user’s interpupillary distance (IPD). In some cases, an inaccurate IPD setting may cause visual distortion and discomfort [43]. Ideally, our test protocol should have included measuring each participant’s IPD and configuring their headset accordingly. However, to simplify the test procedure, we opted to set the IPD to 63 mm, which is the average in adults [44], and only adjusted the IPD setting for users if they reported discomfort or an unacceptable image quality. The participants’ reports of blurred vision may partly be explained by an incorrect IPD setting. Future study protocols should include measuring the participants’ IPD to improve the perceived image quality and reduce the risk of eye strain and discomfort.

### Conclusions

This paper described an iterative user-centered design process for a VNE for older adults. We presented the participants’ preferences and ideas, how these were realized in the ongoing development of a VNE prototype, and how new implementations were received by the participants. We proposed 3 principles for VNEs for older adults: realness, interactivity, and relatedness. We also provided suggestions that can be considered by designers and researchers of VNEs for older adults. These include trueness to reality in terms of rendition and behavior; traces of human activity and natural processes that trigger the imagination and provide believability; the ability to roam, explore, and interact with the environment; and a familiar, relatable environment that evokes memories. VNEs should provide a diversity of content and activities to accommodate the heterogeneity in older adults’ preferences. We argued that non–VR-related theories of restorative natural environments may not be applicable in a VR context. This study can contribute to the development of a framework for designing VNEs for older adults.

## Appendix

Multimedia Appendix 1 Questionnaires used in this study.

## Abbreviations

HMD: head-mounted display
IMI: Intrinsic Motivation Inventory
IPD: interpupillary distance
Psi: plausibility
SUS: System Usability Scale
VNE: virtual natural environment
VR: virtual reality
