# Supplementary material for: Designing Virtual Natural Environments for Older Adults: Think-Aloud Study
Source: JMIR Hum Factors. 2023 Apr 7;10:e40932. doi: 10.2196/40932 (PMC10131598; doi:10.2196/40932)
Supplement: Multimedia Appendix 1 [file humanfactors_v10i1e40932_app1.docx]

# Multimedia appendix 1, Questionnaires

## Intrinsic Motivation Inventory (IMI) questionnaire [37]

All items used a 1-7 Likert scale. Some items were omitted, and the wording was altered slightly, e.g., replacing “this activity” with “the virtual experience”. The items were translated into Swedish. The headings were removed, and the order of the items was randomized.

### Interest/Enjoyment

I enjoyed the virtual experience very much.

This virtual experience was fun to do.

I thought the virtual experience was boring.

The virtual experience did not hold my attention at all.

I would describe the virtual experience as very interesting.

I thought the virtual experience was quite enjoyable.

While I was doing the virtual experience, I was thinking about how much I enjoyed it.

### Pressure/Tension

I felt very tense during the virtual experience.

I was very relaxed during the virtual experience.

I was nervous during the virtual experience.

I felt pressured during the virtual experience.

### Value/Usefulness

I believe the virtual experience could be of some value to me.

I think that the virtual experience is useful for me.

I would be willing to do the virtual experience again because it has some value to me.

I believe the virtual experience is beneficial for me to do.

I believe the virtual experience is important for me to do.

## System Usability Scale (SUS) [35]

All items used a 1-5 Likert scale. The wording was altered slightly, e.g., replacing “this system” with “this” or similar. The items were translated into Swedish.

1. I think that I would like to use this frequently.
2. I found it to be unnecessarily complex.
3. I thought it was easy to use.
4. I think that I would need the support of a technical person to be able to use this.
5. I found the various functions were well integrated.
6. I thought there was too much inconsistency.
7. I would imagine that most people would learn to use this very quickly.
8. I found this very cumbersome to use.
9. I felt very confident while using this.
10. I needed to learn a lot of things before I could get going with this.

## Background questionnaire

The questionnaire was in Swedish but has been translated into English below.

1. **What kind of residence do you live in?
   (apartment/detached house/row house/special accommodations/other)**------------------------------------------------------------------
2. **Do you cohabit or live alone?***Check one box only.*
   ▢ Cohabit
   ▢ Alone
3. **Do you have access to a garden in connection with your residence?***Check one box only.*▢ Yes
   ▢ No
4. **How much experience do you have of using virtual reality?**

*Check one box only.*

1 2 3 4 5

None ▢ ▢ ▢ ▢ ▢ Very much

1. **How much experience do you have of natural environments?**

*Check one box only.*

1 2 3 4 5

None ▢ ▢ ▢ ▢ ▢ Very much

1. **How great are your possibilities to spend time in natural environments?**

*Check one box only.*

1 2 3 4 5

None ▢ ▢ ▢ ▢ ▢ Very great

1. **How often do you spend time in natural environments?**

*Check one box only.*

1 2 3 4 5

Never ▢ ▢ ▢ ▢ ▢ Very often

1. **What are your hobbies?**
   ------------------------------------------------------------------
   ------------------------------------------------------------------
   ------------------------------------------------------------------
2. **What were your hobbies as a child?**------------------------------------------------------------------
   ------------------------------------------------------------------
   ------------------------------------------------------------------
3. **How do you feel?**

*Check one box only.*

1 2 3 4 5

Very bad ▢ ▢ ▢ ▢ ▢ Perfectly good

## Virtual Reality Symptom Questionnaire (VRSQ) [38]

Items 1-13 used a 0-6 rating scale where 0 = “none” and 6 = “severe”. Item 14 was open ended. The questionnaire was translated into Swedish.

### General body symptoms

1. General discomfort
2. Fatigue
3. Boredom
4. Drowsiness
5. Headache
6. Dizziness
7. Difficulty concentrating
8. Nausea

### Eye related symptoms

1. Tired eyes
2. Sore/aching eyes
3. Eyestrain
4. Blurred vision
5. Difficulty concentrating
6. Other symptoms/feelings
